# Survival and morbidity in very preterm infants in Shenzhen: a multi-center study

**DOI:** 10.3389/fped.2023.1298173

**Published:** 2024-02-23

**Authors:** Tingting Li, Guofei Zhang, Rui Li, Shengnan He, Fangshi Zhang, Xudong Yan, Zhangbin Yu, Yingmei Xie, Shenzhen Neonatal Data Network, Guichao Zhong

**Affiliations:** ^1^ Department of Neonatology, Longgang District Maternity & Child Healthcare Hospital of Shenzhen City, Longgang Maternity and Child Institute of Shantou University Medical College, Shenzhen, China; ^2^ Department of Neonatology, Qinghai Red Cross Hospital, Xining, China; ^3^ Department of Neonatology, Shenzhen Baoan Women’s and Children’s Hospital, Jinan University, Shenzhen, China; ^4^ Department of Neonatology, Shenzhen Yantian District People’s Hospital, Shenzhen, China; ^5^ Department of Neonatology, Shenzhen People’s Hospital, Shenzhen, China

**Keywords:** preterm, infants, survival, morbidity, multi-center

## Abstract

**Objective:**

To analyze survival and morbidity among very preterm infants (VPIs) in Shenzhen and explore factors associated with survival without major morbidity.

**Methods:**

Between January 2022 and December 2022, 797 infants were admitted to 25 neonatal intensive care units in Shenzhen with gestational age (GA) < 32 weeks, excluded discharged against medical advice, insufficient information, and congenital malformation, 742 VPIs were included. Comparison of maternal and neonate characteristics, morbidities, survival, and survival without major morbidities between groups used Mann Whitney *U* test and *X*^2^ test, multivariate logistic regression was used to analyze of risk factors of survival without major morbidities.

**Results:**

The median GA was 29.86 weeks (interquartile range [IQR], 28.0–31.04), and the median birth weight was 1,250 g (IQR, 900–1,500). Of the 797 VPIs, 721 (90.46%) survived, 53.52% (38 of 71) at 25 weeks’ or less GA, 86.78% (105 of 121) at 26 to 27 weeks' GA, 91.34% (211 of 230) at 28 to 29 weeks' GA, 97.86% (367 of 375) at 30 to 31 weeks' GA. The incidences of the major morbidities were moderate-to-severe bronchopulmonary dysplasia,16.52% (113 of 671); severe intraventricular hemorrhage and/or periventricular leukomalacia, 2.49% (17 of 671); severe necrotizing enterocolitis, 2.63% (18 of 671); sepsis, 2.34% (16 of 671); and severe retinopathy of prematurity, 4.55% (27 of 593), 65.79% (450 of 671) survived without major morbidities. After adjustment for GA, birth weight, and 5-min Apgar score, antenatal steroid administration (OR = 2.397), antenatal magnesium sulfate administration (OR =  1.554) were the positivity factors to survival without major morbidity of VPIs, however, surfactant therapy (OR = 0.684,), and delivery room resuscitation (OR = 0.626) that were the negativity factors.

**Conclusions:**

The present results indicate that survival and the incidence of survival without major morbidities increased with GA. Further, antenatal administration of steroids and magnesium sulfate, surfactant therapy, and delivery room resuscitation were pronounced determinants of survival without morbidities.

## Introduction

Preterm birth is the leading cause of neonatal mortality, despite a significant increase in the survival rate of preterm infants in recent years (1). Globally, over 15 million preterm babies are born, and China accounts for 7.8% of preterm births (2). The survival rate of preterm infants has significantly increased as a result of the use of antenatal steroids, pulmonary surfactant, and respiratory support procedures, particularly in affluent nations with an abundance of medical resources (3). Statistical data reveal that 95.4% of babies born at gestational age (GA) < 32 weeks survived, whereas only 62.3% of those born at GA <28 weeks survived (1, 4). Thus, it seems that the smaller the GA, the greater is the risk of not surviving. However, short- and long-term complications, such as neurodevelopmental, behavioral, sensory, and respiratory problems, are common among survivors (5). Thus, the key to the successful management of preterm infants is to ensure their survival as well as to prevent any serious morbidities.

Each year, approximately 0.2 million very preterm infants (VPIs) are born in China (6). Although the newborn survival rate has increased dramatically in recent years in China, preterm infants, particularly VPIs, still have a higher rate of survival without serious morbidity than with serious morbidity. It is estimated that the birth rate in Shenzhen has been higher than the national average for the past 5 years, and ranges from 15.09‰ to 21.68‰ (7). A few studies have reported survival and morbidity in preterm infants, but there is very little information about survival without morbidity in preterm infants in Shenzhen. Municipal-level data on survival and short-term complications of VPIs in Shenzhen are lacking. Therefore, this study analyze the data would be useful for analyzing the current situation with regard to the survival of VPIs and improving decision making.

We found that data on survival and morbidity rates for VPIs in Shenzhen were available from the Shenzhen Neonatal Data Network (SNDN), which was launched in June 2022 to collect data on inpatient births. The network includes 26 neonatal intensive care units (NICUs), of which 19 were general hospitals, 7 were specialized hospitals (Supplementary Data Sheet 1). The SNDN database was launched with retrospective data collection starting from January 1, 2022. Data acquisition were abstracted by data abstractors in each hospital, data was collected and transmitted to the SNDN database. The data collected included maternal information, neonatal information, antenatal care, major morbidities, and outcome at discharge. Therefore, a multi-center survey was conducted to collect a series of data from SNDN. Twenty-two hospitals in Shenzhen collected whole-year data of VPIs admitted to their NICUs in 2022 and were enrolled in this study (4 hospitals were excluded because of one was non-Shenzhen hospital, the others data were incomplete for logistical reasons but were included in calculation of survival rates), that investigated the survival and morbidity of VPIs and the associated risk factors. This study aimed to estimate the outcomes of VPIs and provide information that could potentially be used to improve the overall survival of VPIs through enhanced future care designs.

## Methods

### Research participants

Data for this multi-center study were obtained from the SNDN database on VPIs with GA <32 weeks who were admitted to the NICUs between 1st January 2022 and 31st December 2022. The inclusion criteria were: GA <32 weeks; admission to the NICU within 24 h after birth; availability of complete clinical data. Infants born with severe congenital malformations or genetic disorders were excluded. The VPIs were divided into eight groups according to GA: <25 weeks, 25 weeks, 26 weeks, 27 weeks, 28 weeks, 29 weeks, 30 weeks, and 31 weeks. Shenzhen People's Hospital Ethics Committee approved this study (approval no. LL- KY-2022288).

### Data collection

Data on the following variables were extracted from data deposited in the SNDN database for VPIs with GA <32 weeks that met the study criteria: maternal information: age, antenatal care, gestational diabetes, antenatal corticosteroids, full course of antenatal corticosteroids, antenatal magnesium sulfate, reproduction status, multiple pregnancy status, chorioamnionitis, duration of premature rupture of membranes, and mode of delivery; neonatal information: GA at birth, birth weight, sex, 1-min and 5-min Apgar scores, delivery room resuscitation, admission hypothermia, application of pulmonary surfactants; and short-term outcomes, including discharge outcomes, ≥grade 3 intraventricular hemorrhage and/or periventricular leukomalacia (IVH/PVL), moderate-to-severe bronchopulmonary dysplasia (BPD), ≥grade 2 necrotizing enterocolitis (NEC), sepsis, and ≥ grade 3 retinopathy of prematurity (ROP).

### Definitions

In this study, a survivor was defined as a neonate who survives to discharge. Neonatal morbidity refers to morbidities that occurred both during the hospital stay and discharge. Hypothermia refers to low body temperature (<36.5°C) recorded at the time of admission of the neonates. Gestational diabetes was defined as diabetes that occurs during pregnancy and included diabetes of all types and levels of severity. Antenatal corticosteroid use meant that the mother had received at least one dose of corticosteroids intravenously or intramuscularly at any time before delivery, and it was defined as a full course of antenatal corticosteroids, which is dexamethasone, if the mother received four intramuscular injections of 6 mg each 12 h apart. The serious morbidities included severe neurological injury, NEC (grade ≥ 2), sepsis, moderate-to-severe BPD, and severe ROP (grade ≥ 3). Severe neurological injury was considered as IVH grade 3 or 4, according to Papile's criteria, or PVL grade 4 (8). NEC at stage 2 or higher was considered as a morbidity based on Bell's criteria (9). Sepsis was defined based on positive blood cultures or cerebrospinal fluid cultures (10). BPD was defined as the need for oxygen or ventilation at 28 days after birth, and moderate-to-severe BPD was defined as the need for oxygen or ventilation at 36 weeks of age when corrected, or at discharge, transfer, or death before 36 weeks (11). Severe ROP was defined as ROP stage 3 or higher or ROP that necessitated therapy (12). Survival without major morbidity was defined as survival in the absence of any of the serious morbidities indicated earlier (13).

### Statistical analysis

SPSS, version 23.0, was used for all the statistical analyses. According to their distribution, continuous variables were presented as the median [interquartile range (IQR)] or the mean and standard deviation (SD). The Pearson chi-squared test was used to compare categorical variables. The median test or Kruskal–Wallis test was used to compare continuous variables. Logistic regression was used in multivariate studies to examine the risk factors for survival without significant morbidity. Results were considered significant at *P* < 0.05.

## Results

### Demographic and clinical information

**Figure 1:**
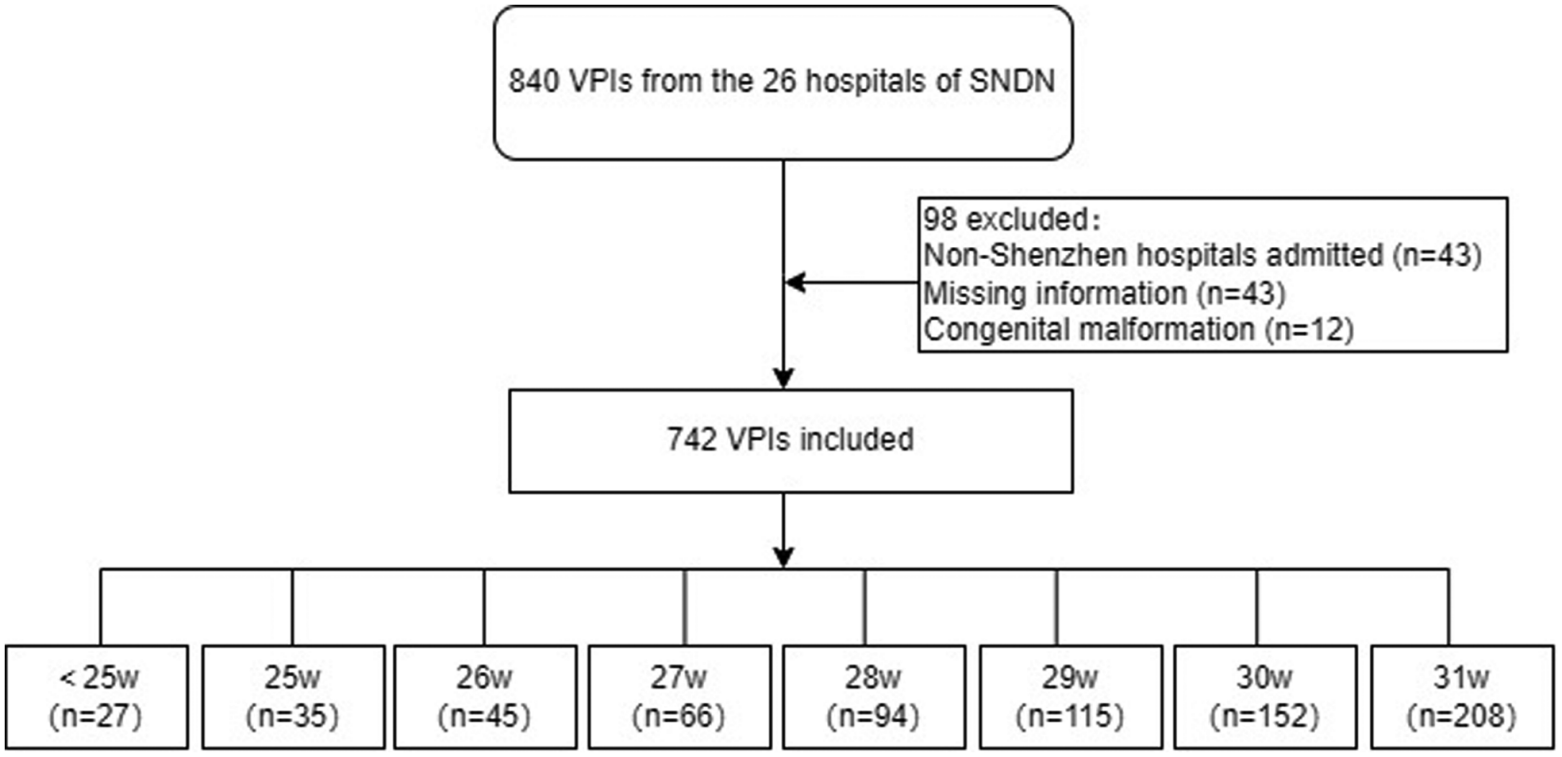
Diagram depicting the protocol of the study. Selection of the VPI cohort analyzed in the study, using data from the database of the Shenzhen Neonatal Digital Network during the period 1st January 2022 to 31st December 2022.

Out of 840 VPIs with recorded data in the SNDN database during the study period, 742 met the inclusion criteria (Figure 1). These VPIs were from 25 NICUs that were part of 15 general hospitals and 7 specialized hospitals. The remaining 98 VPIs were excluded because of discharged against medical advice (DAMA) (*n* = 40), insufficient information (*n* = 3), congenital abnormalities (*n* = 12), and admission to 2 hospitals outside of Shenzhen (*n* = 43). The median GA and birth weight of the 742 included VPIs were 29.86 (28.00–31.04) weeks and 1,250 (990–1,500) g, respectively (Table 1). Among the eight groups based on GA, a significant difference was found in the incidence of GDM, the administration of antenatal steroids, magnesium sulfate therapy, the use of assisted reproductive therapy, cesarean section, chorioamnionitis, birth weight, multiple births, the number of neonates with 1- and 5-min Apgar score ≥ 7, delivery room resuscitation, and administration of surfactant therapy (*P* < 0.05).

**Table 1:** Maternal and neonate characteristics in the current cohort of very preterm infants (<32 gestational weeks) admitted to 25 NICUs in Shenzhen, China.

Characteristic		No./Total No. (%)						
	Total	Gestational age, wk						
		<24 w	24–25 w	26–27 w	28–29 w	30–31 w	*X*^2^/z	*P*值
Maternal characteristic								
No.	742	16	52	122	192	360		
Age, y, M (Q1,Q3)	31 (28,35)	32 (30, 36)	32 (28, 34.75)	32 (29, 35)	31 (28, 34)	31 (28, 35)	2.57	0.63
≥1 antenatal care visit, *n* (%)	727 (98.00)	16 (100)	50 (96.15)	121 (99.18)	189 (98.44)	351 (97.5)	2.714	.607
Gestational diabetes, *n* (%)	200 (27.00)	0 (0)	9 (17.31)	26 (21.31)	44 (22.92)	121 (33.61)	20.027	0.00
ANS, *n* (%)	673 (90.70)	12 (75)	42 (80.77)	114 (93.44)	176 (91.67)	329 (91.39)	12.259a	0.016
Full course of ANS, *n* (%)	376 (50.67)	3 (18.75)	29 (55.77)	66 (54.1)	103 (53.65)	175 (48.61)	8.927a	.063
Antenatal magnesium sulfate, *n* (%)	559 (75.34)	11 (68.75)	42 (80.77)	95 (77.87)	152 (79.17)	259 (71.94)	5.37	0.25
Assisted reproduction status, *n* (%)	172 (23.18)	5 (31.25)	17 (32.69)	36 (29.51)	42 (21.88)	72 (20)	8.199a	0.09
Cesarean delivery, *n* (%)	504 (67.92)	3 (18.75)	17 (32.69)	81 (66.39)	143 (74.48)	260 (72.22)	54.35	0.00
PROM > 18 h, *n* (%)	36 (4.85)	0 (0)	3 (5.77)	9 (7.38)	9 (4.69)	15 (4.17)	2.97	0.56
Chorioamnionitis, *n* (%)	357 (48.11)	12 (75)	30 (57.69)	75 (61.48)	100 (52.08)	140 (38.89)	28.75	0.00
Infant characteristics								
Gestational age, wk, M(Q1,Q3)	29.86 (28.00,31.04)	22.86 (22.04, 23.57)	25.36 (25, 25.82)	27.43 (26.86, 27.86)	29 (28.57, 29.43)	31.14 (30.57, 31.57)	640.54	0.00
Birth weight, M(Q1,Q3)	1,250 (990,1,500)	500 (411.25, 589.25)	805 (695, 850)	954 (897.5, 1,032.5)	1,200 (1,092.5, 1,300)	1,500 (1,380, 1,658.75)	465.03	0.00
Male, *n* (%)	347 (46.77)	8 (50)	15 (28.85)	60 (49.18)	102 (53.13)	162 (45)	13.931	0.05
Multiple birth, *n* (%)	230 (30.99)	10 (62.5)	20 (38.5)	41 (33.6)	55 (28.6)	104 (28.9)	10.411	0.034
1-min APGAR score ≤7, *n* (%)	163 (21.97)	12 (75)	27 (51.92)	48 (39.34)	40 (20.83)	36 (10)	105.184	0.000
5-min APGAR score ≤7, *n* (%)	69 (9.30)	10 (62.5)	14 (26.92)	16 (13.11)	18 (9.38)	11 (3.06)	91.59	0.00
Delivery room resuscitation, *n* (%)	559 (75.34)	16 (100)	49 (94.23)	109 (89.34)	153 (79.69)	232 (64.44)	53.055a	0.00
Admission hypothermia, *n* (%)	436 (58.76)	10 (62.5)	34 (65.38)	83 (68.03)	98 (51.04)	211 (58.61)	10.09	0.04
Surfactant therapy, *n* (%)	387(52.16)	10(62.5)	38(73.08)	89(72.95)	116(60.42)	134(37.22)	68.37	.000

ANS, antenatal corticosteroids.

### Survival and morbidities

The total incidence of ≥ grade 3 IVH or PVL, moderate-to-severe BPD, ≥grade 2 NEC, sepsis, and ≥ grade 3 ROP was 2.49%, 16.52%, 2.63%, 2.34%, and 4.55%, respectively. The incidence of major morbidities decreased as GA increased (Table 2). The survival rate of the VPIs was 90.46% (721 out of 797). With regard to GA, the survival rates for <25-, 25-, 26-, 27-, 28-, 29-, 30-, and 31-week VPIs were 24.14%, 73.81%, 84.91%, 88.24%, 90.91%, 92.37%, 97.53%, and 98.12%, respectively (Table 3). For all the VPIs, the survival rate without serious morbidity was 65.79% (450 of 671). With increase in GA, the rates of survival and survival without major morbidities increased. The rate of survival was no significant differences between general hospitals and specialize hospitals (*P* > 0.05), it is that higher survival without major morbidities in the specialized hospitals than general hospitals (*P* < 0.05) (Supplementary Table 1 in the Supplementary Material).

**Table 2:** Morbidities in very preterm infants (<32 gestational weeks) admitted to 25 NICUs.

Variable	Infants, No./Total No. (%)										
	Total	Gestational age, wk									
		<25 w	25 w	26 w	27 w	28 w	29 w	30 w	31 w	*X* ^2^	*P*
	671	7	25	38	56	85	111	143	206	-	-
≥3 grade IVH /PVL	17 (2.49)	1 (14.29)	3 (11.54)	3 (7.89)	4 (7.02)	2 (2.27)	3 (2.65)	0 (0)	1 (0.48)	-	0.00*
Moderate-to-severe BPD	113 (16.52)	6 (85.71)	19 (73.08)	15 (39.47)	21 (36.84)	13 (14.77)	10 (8.85)	10 (6.76)	19 (9.18)	140.597	0.00
NEC stage ≥2	18 (2.63)	0 (0)	1 (3.85)	4 (10.53)	1 (1.75)	1 (1.14)	6 (5.31)	3 (2.03)	2 (0.97)	-	0.035*
Sepsis	16 (2.34)	0 (0)	2 (7.69)	2 (5.26)	3 (5.26)	1 (1.14)	5 (4.42)	1 (0.68)	2 (0.97)	13.244	0.031
ROP stage ≥3^a^	27/593 (4.55)	4/7 (57.14)	8/20 (40)	5/35 (14.29)	7/53 (13.21)	1/76 (1.32)	0/101(0)	1/124(0.81)	1/177(0.56)	-	0.00*

BPD, bronchopulmonary dysplasia; IVH, intraventricular hemorrhage; NEC, necrotizing enterocolitis; NICU, neonatal intensive care unit; PVL, periventricular leukomalacia; ROP, retinopathy of prematurity.

^a^ ROP was evaluated among infants with eye examinations.

* Significant at *P* < 0.05, as determined by the Fisher test.

**Table 3:** Survival and survival without morbidities in very preterm infants (<32 gestational weeks) admitted to 25 NICUs.

Variable	Infants, No./total No. (%)								
	Total	Gestational age, wk							
		<25 w	25 w	26 w	27 w	28 w	29 w	30 w	31 w
Survival^a^	721/797	7/29	31/42	45/53	60/68	90/99	121/131	158/162	209/213
	(90.46)	(24.14)	(73.81)	(84.91)	(88.24)	(90.91)	(92.37)	(97.53)	(98.12)
Survival without major morbidity^b^	450/671 (65.79)	0/7 (0)	3/25 (11.54)	9/38 (23.68)	24/56 (42.11)	58/85 (65.91)	81/111 (71.68)	112/143 (75.68)	163/206 (78.74)

^a^ Calculated among VPIs who were admitted to NICU, included for discharge against medical, insufficient information, congenital malformation and survived to discharge.

^b^ Calculated among VPIs who excluded for discharge against medical, insufficient information and congenital malformation.

### Multivariate analysis of the incidence of survival without major morbidities

**Figure 2:**
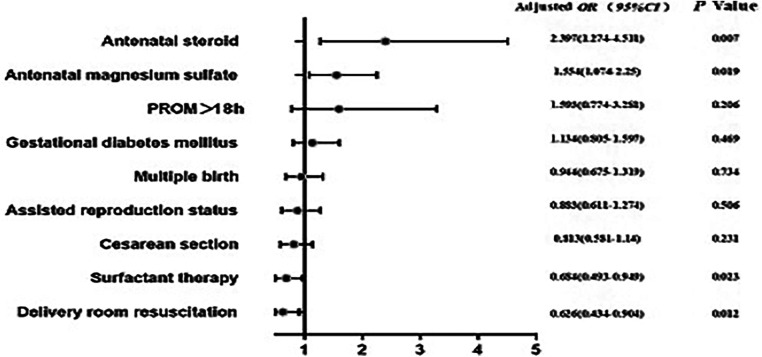
Multivariate logistic regression analysis of risk factors for survival without major morbidity. Antenatal steroid administration, magnesium sulfate treatment, surfactant therapy, and resuscitation in the delivery room were identified as significant indicators of survival without severe morbidity. #*P* values after adjusting for gestational age, birth weight, and 5-min Apgar score.

According to the multivariable logistic regression model, after adjusting for GA, birth weight, and the 5-min Apgar score, survival of VPIs without major morbidity was found to be significantly associated with the use of antenatal steroids (OR = 2.397, 95% CI—1.274–4.511), magnesium sulfate use (OR = 1.554, 95% CI = 1.074–2.250), surfactant therapy (OR = 0.684, 95% CI = 0.493–0.949), and delivery room resuscitation (OR = 0.626, 95% CI = 0.434–0.904) (Figure 2).

## Discussion

The present study examines survival and morbidity in a cohort of VPIs from the Shenzhen region of China, and also identifies factors associated with survival without major morbidities. To our knowledge, this study included 742 VPIs admitted to NICUs from 25 hospitals that have data deposited in SDND, that is the first municipal-level comprehensive assessment of survival and morbidities of very preterm infants in NICUs and serves to fill a gap in our knowledge of the current status of neonatal in Shenzhen. The results is based on benchmark outcomes for hospital to evaluate their performance, facilitate quality improvement and support parental counseling and clinical decision-making.

The survival rate of VPIs was 90.46% among all infants admitted to NICU. The data revealed that the survival rate increased with increase in GA. That is, the survival rate for VPIs improved from 24.14% at <25 weeks GA to 98.12% at 31 weeks GA. DAMA, insufficient information and congenital malformation substantially compromised the survival of VPIs. Therefore, we studied survival among the VPIs (Table 3). These results are in accordance with those reported by Zhu et al. (1), who reported survival rates for infants with complete care in 68 Chinese NICUs in 2010–2019 of 62.30% vs. 74.48% for extremely preterm, those with GA less than 28 weeks. In comparison to earlier data from China (14, 15), our findings imply a substantial rise in survival rates. Our results, however, still fall short of those reported by prestigious hospitals from more developed countries. For example, According to Cao et al. (4), 9,442 VPIs from 57 tertiary institutions in China had a 95.4% survival rate. The higher rate may be attributable to better medical facilities at these hospitals. Another one, the findings of a large international cohort study (16) on 88,327 preterm infants from neonatal collaborative networks in ten developed nations revealed that the overall survival rate of preterm infants at 24–29 weeks was 87%, with Japan having the highest survival rate (93%) and Spain having the lowest (78%).

Our investigation found an overall prevalence of 2.49%, 16.52%, 2.63%, 2.34%, and 4.55% for ≥ grade 3 IVH and/or PVL, moderate-to-severe BPD, ≥stage 2 NEC, sepsis, and ≥ grade 3 ROP, respectively. According to the findings, moderate-to-severe BPD continues to be the most common severe outcome of VPIs in Shenzhen, accounting for the majority of the morbidities. Similarly, according to the neonatal research networks of Japan and Brazil, the reported prevalence of BPD in newborns is high at 18.2% and 19.2%, respectively (17). Our finding of 90.70% antenatal corticosteroid use is the potential reasons, antenatal corticosteroid have been demonstrated to improve neonatal outcomes in preterm infants. According to reports, the prevalence of NEC in VPIs ranges from 3.1% to 8.8% in middle- and high-income countries (18–21). However, the incidence of sepsis in VPIs from the present cohort (2.63%) is lower than that reported in the Netherlands (22) (15.9%). This difference could primarily be attributed to differences in the comprehensive management of VPIs by various collaborating units. However, it could also indicate a lower rate of diagnosis of sepsis or detection of causative microbes. Thus, this finding may not necessarily imply higher levels of sepsis control at the investigated NICUs and warrants further investigation. In a French study that included VPIs, higher prevalence of severe IVH (5.3%) and lower prevalence of severe ROP (1.2%) were found (23). In another cohort study from China, the prevalence of severe ROP and severe brain injury is 10.4% and 4.3%, respectively (4). Major morbidity is associated with long-term developmental results, so even more crucial than survival is major morbidity-free survival. The rates of survival without major morbidities are still lower than those noted in nations with developed medical systems (16). Reducing morbidities and raising quality of life will become more crucial as survival rates rise, therefore newborn follow-up, early intervention, and developmental care should be developed adequately in Shenzhen. In this case, the survival without major morbidities in the specialized hospitals is higher than the general hospitals (*P* < 0.05), it indicated that specialized hospitals may have more mature experience. Antenatal steroid and antenatal magnesium sulfate were found to be significant determinants of survival without major morbidity, according to the results of logistic regression analysis conducted in the present study. This finding corresponds well with that reported in Ethiopia (24), another study from China (4), and Austria (25). This is probably related to the positive effect of antenatal steroid and magnesium sulfate treatment on fetal maturity and immunity, as this could have led to a reduction in the number of complications caused by prematurity. The present study also found that surfactant therapy and resuscitation in the delivery room were negativity factors as significant predictors of survival without major morbidities. Similarly, a meta-analysis on 30 studies reported that the application of surfactant therapy could imply neonate short- and long-term outcomes (26). Typically, resuscitation in the delivery room is the primary treatment for asphyxia. Under conditions of asphyxia, the fetus attempts to redistribute cardiac output to protect more crucial organs, such as the brain, heart, and adrenal gland, at the expense of decreasing blood flow to other organs such as the gut and the lungs (27). This may increase their chances of survival without morbidity in the neonate, but the chances of pulmonary morbidities can be increased with delivery room resuscitation and surfactant treatment, as demonstrated by the present results.

In order to accurately reflect the treatment of VPIs in Shenzhen, this study covered 25 hospitals in Shenzhen, including 18 general hospitals and 7 specialized hospitals. The hospitals studied provided different levels of treatment and were, therefore, representative of different hospital systems. The study does, however, have several limitations that must be noted, and the results must be carefully interpreted in light of these limitations. (1) The majority of the reported morbidities included in this study was reported in VPIs who survived. As the mortality rate was low, this could have introduced a bias in the analysis. (2) We did not specifically consider factors that have been attributed to mortality in VPIs, as this could have generated a bias. (3) This study is hospital-based, rather than population-based, so our sample may not adequately represent the preterm population in the country.

## Conclusions

The overall survival rate for very preterm newborns was 90.46%, and 65.79% of them survived without experiencing significant problems. Based on the factors that were found to be associated with survival without morbidities, it is recommended that prenatal corticosteroids, magnesium sulfate to be essential for the survival of VPIs without morbidity. Overall, the findings imply the need for more aggressive and efficient treatment approaches, particularly for infants born at GA ≤25 weeks.

## Data Availability

The original contributions presented in the study are included in the article/Supplementary Material, further inquiries can be directed to the corresponding authors.

## Group members of Shenzhen Neonatal Data Network

Guichao Zhong: Shenzhen People's Hospital; Cheng Chen: Longgang District Maternity & Child Healthcare Hospital of Shenzhen City (Longgang Maternity and Child Institute of Shantou University Medical College); Xueling Zhuang: Shenzhen Baoan Women's and Children's Hospital, Jinan University; Yanping Guo: Peking University Shenzhen Hospital; Huixian Qui: Longgang District Central Hospital of Shenzhen; Qianshen Zhang: The University of Hongkong, Shenzhen Hospita; Jinxing Feng: Shenzhen Children's Hospital; Ya Pan: Shenzhen Longhua Maternity and Child Healthcare Hospital; Jiaoyu Mao: Huazhong University of Science and Technology Union Shenzhen Hospital; Rui Wang: Shenzhen Luohu Hospital Group Luohu People's Hospital; Gerun Zhang: Seventh Affiliated Hospital, Sun Yat-sen University; Qianqian Fan: Shenzhen Longhua District Central Hospital; Xiaoli Chen: University of Chinese Academy of Sciences-Shenzhen Hospital; Yuefeng Li: Shenzhen Luohu Maternity and Child Healthcare Hospital; Xiaoguang Zhou: The Eighth Affiliated Hospital, Sun Yat-sen University; Erya Ying: Shenzhen Nanshan District Maternity & Child Healthcare Hospital; Zhihong Zhong: Shenzhen Hospital of Integrated Traditional Chinese and Western Medicine; Binyu Ni: Longgang District People's Hospital of Shenzhen; Yanrong Wang: The Third People's Hospital of Shenzhen; Hong Tong: Shenzhen Yantian District People's Hospital. Gang Liu: ShenZhen Pingshan Maternal and Child Health Hospital, Shenzhen, China.
