# Supplementary material for: Survival and morbidity in very preterm infants in Shenzhen: a multi-center study
Source: Front Pediatr. 2024 Feb 23;11:1298173. doi: 10.3389/fped.2023.1298173 (PMC10920349; doi:10.3389/fped.2023.1298173)
Supplement: Supplementary file 1 [file Datasheet1.pdf]

Supplement 1

Shenzhen Neonatal Data Network

The Shenzhen Neonatal Data Network consists of these participating hospitals:Shenzhen People's Hospital,Shenzhen,China;Longgang District Maternity & Child Healthcare Hospital of Shenzhen City (Longgang Maternity and Child Institute of Shantou University Medical College) ,Shenzhen,China;Shenzhen Baoan Women's and Children's Hospital, Jinan University,Shenzhen,China;Peking University Shenzhen Hospital,Shenzhen,China;Longgang District Central Hospital of Shenzhen,Shenzhen,China;People's Hospital of Longhua, Shenzhen,China;The University of Hongkong, Shenzhen Hospital,Shenzhen,China;Shenzhen Children's Hospital,Shenzhen,China;Shenzhen Longhua Maternity and Child Healthcare Hospital,Shenzhen,China;Huazhong University of Science and Technology Union Shenzhen Hospital,Shenzhen,China;Shenzhen Luohu Hospital Group Luohu People's Hospital,Shenzhen,China;Seventh Affiliated Hospital, Sun Yat-sen University,Shenzhen,China;Shenzhen Longhua District Central Hospital,Shenzhen,China;University of Chinese Academy of Sciences-Shenzhen Hospital,Shenzhen,China;Shenzhen Luohu Maternity and Child Healthcare Hospital,Shenzhen,China;The Eighth Affiliated Hospital, Sun Yat-sen University,Shenzhen,China;Shenzhen Nanshan District Maternity & Child Healthcare Hospital,Shenzhen,China;Shenzhen Hospital of Integrated Traditional Chinese and Western Medicine,Shenzhen,China;Longgang District People's Hospital of Shenzhen,Shenzhen,China;ShenZhen Pingshan Maternal and Child Health Hospital,Shenzhen,China;The Third People's Hospital of Shenzhen,Shenzhen,China;Shenzhen Yantian District People's Hospital,Shenzhen,China.
