# Supplementary material for: Survival and morbidity in very preterm infants in Shenzhen: a multi-center study
Source: Front Pediatr. 2024 Feb 23;11:1298173. doi: 10.3389/fped.2023.1298173 (PMC10920349; doi:10.3389/fped.2023.1298173)
Supplement: Supplementary file 2 [file Table1.pdf]

Supplementary Table 1. Survival and survival without major morbidity of VPIs admitted to different type of hospitals

	Infants，No./total No.(%)				
		Type of Hospital			
**Variable**	**Total**	**General hospitals**	**Specialized hospitals**	**X^2**	**P**
**Survival[a]（n=797）**					
**YES**	**721**	**432（59.92）**	**289（40.08）**	**0.597**	**0.440**
**NO**	**76**	**49（64.47）**	**27（35.53）**		
**Survival without major morbidity[b]（n=721）**					
**YES**	**450**	**291（64.67）**	**159（35.33）**	**11.247**	**0.001**
**NO**	**271**	**141（52.03）**	**130（47.97）**		

[a] Calculated among VPIs who were admitted to NICU, included for discharge against medical, insufficient information, congenital malformation.

b Calculated among VPIs survived to discharge who excluded for discharge against medical, insufficient information and congenital malformation.
